# Antioxidant Activity and DPP-IV Inhibitory Effect of Fish Protein Hydrolysates Obtained from High-Pressure Pretreated Mixture of Rainbow Trout (*Oncorhynchus mykiss*) and Atlantic Salmon (*Salmo salar*) Rest Raw Material

**DOI:** 10.3390/md22120568

**Published:** 2024-12-18

**Authors:** Elissavet Kotsoni, Egidijus Daukšas, Grete Hansen Aas, Turid Rustad, Brijesh K. Tiwari, Carmen Lammi, Carlotta Bollati, Melissa Fanzaga, Lorenza d’Adduzio, Janne Kristin Stangeland, Janna Cropotova

**Affiliations:** 1 Department of Biological Sciences Ålesund, Norwegian University of Science and Technology, 6009 Ålesund, Norway; egidijus.dauksas@ntnu.no (E.D.); graa@ntnu.no (G.H.A.); 2 Department of Biotechnology and Food Science, Norwegian University of Science and Technology, 7034 Trondheim, Norway; turid.rustad@ntnu.no; 3 Food Chemistry and Technology Department, Teagasc Food Research Centre, Ashtown, D15 DY05 Dublin, Ireland; brijesh.tiwari@teagasc.ie; 4 Department of Pharmaceutical Sciences, Università degli Studi di Milano, Via Luigi Mangiagalli 25, 20133 Milano, Italy; carmen.lammi@unimi.it (C.L.); carlotta.bollati@unimi.it (C.B.); melissa.fanzaga@unimi.it (M.F.); lorenza.dadduzio@unimi.it (L.d.); 5 Møreforsking AS, Borgundvegen 340, 6009 Ålesund, Norway; janne.kristin.stangeland@moreforsking.no

**Keywords:** innovative technologies, fish side streams, molecular weight distribution, bioactive peptides, Caco-2 cells

## Abstract

The use of fish rest raw material for the production of fish protein hydrolysates (FPH) through enzymatic hydrolysis has received significant interest in recent decades. Peptides derived from fish proteins are known for their enhanced bioactivity which is mainly influenced by their molecular weight. Studies have shown that novel technologies, such as high-pressure processing (HPP), can effectively modify protein structures leading to increased biological activity. This study investigated the effect of various HPP conditions on the molecular weight distribution, antioxidant activity, and dipeptidyl-peptidase IV (DPP-IV) inhibitory effect of FPH derived from a mixture of rainbow trout (*Oncorhynchus mykiss*) and Atlantic salmon (*Salmo salar*) rest raw material. Six different treatments were applied to the samples before enzymatic hydrolysis; 200 MPa × 4 min, 200 MPa × 8 min, 400 MPa × 4 min, 400 MPa × 8 min, 600 MPa × 4 min, and 600 MPa × 8 min. The antioxidant and DPP-IV inhibitory effects of the extracted FPH were measured both in vitro and at cellular level utilizing human intestinal Caco-2 cells. The results indicated that low and moderate pressures (200 and 400 MPa) increased the proportion of larger peptides (2–5 kDa) in the obtained FPH, while treatment at 600 MPa × 4 min resulted in a higher proportion of smaller peptides (1–2 kDa). Furthermore, HPP led to the formation of peptides that demonstrated increased antioxidant activity in Caco-2 cells compared to the control, whereas their potential antidiabetic activity remained unaffected.

## 1. Introduction

The growing global population, expected to exceed 9 billion by 2050 [1], has increased the demand for sustainable and more efficient utilization of food resources. Seafood stands out as an excellent source of nutrients, being rich in high-quality proteins, vitamins, minerals and n-3 polyunsaturated fatty acids (n-3 PUFA), and containing low levels of saturated fat [2]. In 2022, seafood accounted for 15% of the animal protein supply and 6% of total protein consumption worldwide [3].

Aquatic food processing generates significant amount of fish rest raw material with a high nutritional value. This includes heads, tails, frames, trimmings, bones, skin, and belly flaps, comprising up to 70% of the processed fish [4]. Recent advances in biotechnology present a promising opportunity to convert this underutilized fish rest raw material—typically discarded, used as fertilizer, or incorporated into animal feed—into high-value products by recovering compounds, such as n-3 PUFAs and bioactive peptides [4,5]. Given the extensive farming of Atlantic salmon (*Salmo salar*) and rainbow trout (*Oncorhynchus mykiss*), efficient utilization of their rest raw material is crucial to managing the large volumes of rest raw material generated [6].

Fish protein hydrolysates (FPH) are derived from the enzymatic hydrolysis of fish raw material, typically using exogenous enzymes [7]. This process breaks down fish proteins into smaller peptides and free amino acids, which demonstrate enhanced bioactivity—especially after fractionation and purification steps—offering significant pharmaceutical and nutraceutical potential [8,9]. The bioactive peptides within FPH are characterized by low molecular weight (2–20 amino acids) and are inactive in the native protein [10]. However, enzymatic hydrolysis releases these peptides, enabling them to demonstrate various properties, including antihypertensive, antioxidant, antimicrobial, anticoagulant, antitumor and immunomodulatory activity [11,12,13]. Among these, antihypertensive and antioxidant activities have been the most extensively studied [14].

The bioactivity of peptides is mainly influenced by their structure, amino acid composition, sequence, and charge [10,12]. Peptides derived from fish proteins have demonstrated antidiabetic activity by inhibiting dipeptidyl-peptidase IV (DPP-IV), that could contribute to the regulation of blood glucose levels [15]. Additionally, antioxidant peptides can scavenge free radicals, implicated in several chronic diseases such as diabetes, cancer, inflammation, and neurodegenerative disorders [16]. Some peptides can also function as metal chelators, reducing oxidative stress by binding prooxidant metal ions [17]. Therefore, the antioxidant activity of peptides in FPH could inhibit lipid oxidation in fortified food systems, thereby improving their shelf life and nutritional quality [18].

High-pressure processing (HPP) is an innovative, non-thermal technology widely used in the seafood industry to preserve and extend their shelf-life without affecting their nutritional value [19]. Studies have shown that combining hydrolysis with HPP, either as pretreatment or concurrently with hydrolysis, the yield and bioactivity of peptides may be improved [20,21,22]. More specifically, HPP can induce conformational changes in proteins, exposing hydrophobic regions and providing more cleavage sites for the enzymes leading to enhanced hydrolysis and peptides with higher bioactivity [21].

The aim of this study was to investigate how varying HPP conditions applied to a mixture of rainbow trout and Atlantic salmon rest raw materials, prior to enzymatic hydrolysis, influenced the bioactive properties of the resulting FPH. Specifically, the study focused on evaluating the antioxidant activity of the FPH using different in vitro assays and in human intestinal Caco-2 cells following H_2_O_2_-induced oxidative stress. Additionally, the anti-diabetic activity of FPH was assessed by measuring their DPP-IV inhibitory effect both in vitro on the human recombinant DPP-IV enzyme and in human intestinal Caco-2 cells.

## 2. Results and Discussion

### 2.1. Molecular Weight Distribution of Peptides

**Figure 1:**
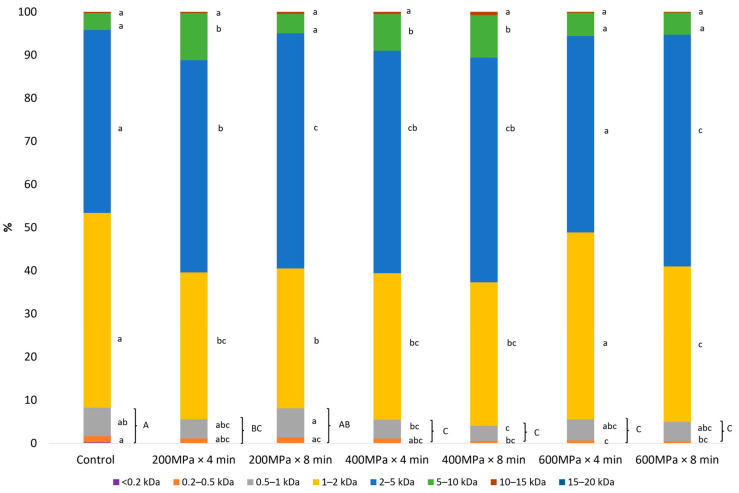
Molecular weight distribution of FPH obtained through enzymatic hydrolysis on samples consisting of a mixture of rainbow trout and Atlantic salmon rest raw material pretreated by HP and on the untreated sample (%, mean, *n* = 3). Distinct lowercase letters denote significant differences among the mean values for the same intervals across various treatment groups. Distinct uppercase letters denote significant differences among the mean values for peptides below 1 kDa across various treatment groups.

The applied HPP conditions exhibited a significant influence on the size of the peptides in the resulting FPH. The obtained FPH from the untreated sample was mainly dominated by peptides with molecular weights in the ranges of 1–2 kDa (45.1%) and 2–5 kDa (42.5%). Smaller amounts of peptides were detected in 0.5–1 kDa (6.6%) and 5–10 kDa (3.9%) ranges, as illustrated in Figure 1. Moreover, moderate (400 MPa) and high (600 MPa) pressure levels produced FPH with lower amounts of peptides smaller than 1 kDa compared to the untreated FPH. Since peptides below 1 kDa are often associated with bitterness in FPH, these findings suggest that HPP may result in FPH with potentially lower bitter taste [23,24]. FPH from samples pretreated by high-pressure (HP) demonstrated a significant reduction in the proportion of peptides in the 1–2 kDa range and a simultaneous increase in peptides in the 2–5 kDa and 5–10 kDa ranges, particularly at low (200 MPa) and moderate pressures (400 MPa). However, the peptide distribution in the FPH from the sample pretreated at 600 MPa × 4 min resembled that of the untreated, showing a higher proportion of smaller peptides (1–2 kDa) compared to samples pretreated at lower pressures. The increased proportion of higher molecular weight peptides at pressures below 400 MPa can be attributed to the increased number of hydrogen bonds, which may lead to increased peptide aggregation [10]. Additionally, the extensive unfolding of proteins induced at higher pressures offers more cleavage sites for subsequent enzymatic breakdown [25]. The increased solubility of protein aggregates that may be observed at higher pressures (600 MPa) [26] along with the initial extensive unfolding could allow the enzymes to access and hydrolyze exposed peptide bonds, thereby producing smaller peptides [10,27]. This hypothesis is supported by the higher degree of hydrolysis (DH%) exhibited by this FPH obtained from the sample pretreated at 600 MPa × 4 min compared to the rest of the samples, as reported in a previous study by Kotsoni, et al. [28]. However, extending the duration to 8 min at 600 MPa, the peptides may have had more time to aggregate and form larger fragments, as evidenced by the significantly higher proportion in the 2–5 kDa range (53.8%) compared to that observed in the FPH obtained from sample subjected to pretreatment at 600 MPa for 4 min (45.5%). This may suggest that prolonged HP pretreatment could promote further aggregation, limiting enzyme accessibility to peptide bonds and resulting in the production of larger peptides (2–5 kDa).

### 2.2. In Vitro Antioxidant Activity Evaluated with Erric Feducing Antioxidant Power (FRAP), 2,2-Azino-Bis-(3-Ethylbenzothiazoline-6-Sulfonic Acid (ABTS), Oxygen Radical Absorbance Capacity (ORAC), and 2,2-Diphenyl-1-Picrylhydrazyl (DPPH) Assays

The in vitro antioxidant capacity of FPH obtained from untreated and HP pretreated samples was evaluated at various concentrations using the FRAP, ABTS, ORAC and DPPH assays. These results were then compared with FPH from samples pretreated by HP at the highest concentrations.

**Figure 2:**
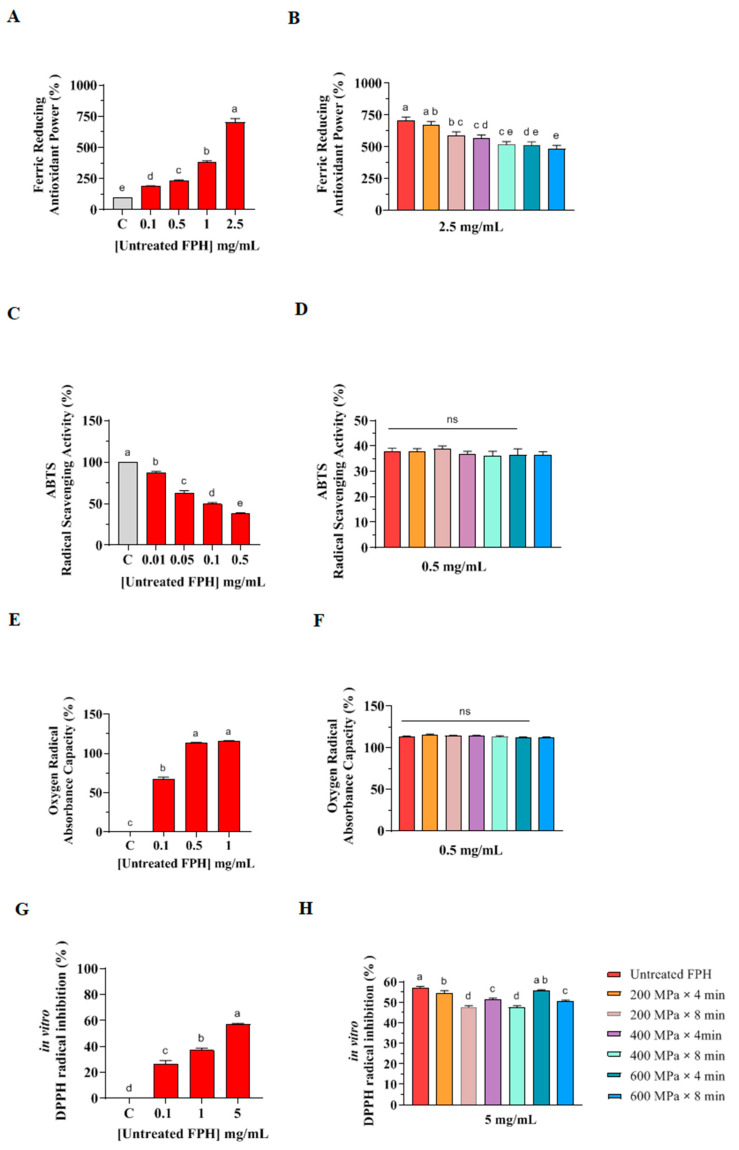
In vitro antioxidant power evaluation of FPH extracted from the control and from samples subjected to HP pretreatment by FRAP (**A**,**B**), ABTS (**C**,**D**), ORAC (**E**,**F**) and DPPH (**G**,**H**) assays. Data represent the mean ± SD, *n* = 3 of four independent experiments. Ordinary one-way ANOVA analysis with multiple comparisons was applied. In graphs (**A**,**C**,**E**,**G**), the FPH from the untreated sample was compared with the vehicle (C) at different concentrations. In graphs (**B**,**D**,**F**,**H**), FPH from samples pretreated by HP were compared with the untreated at the highest concentration. C: control sample (H_2_O); ns: not significant. Distinct lowercase letters denote significant differences among the mean values across various treatment groups.

As shown in Figure 2 (see also Table S1 in Supplementary Materials), the untreated FPH sample demonstrated antioxidant capacity in all the antioxidant experiments conducted. In particular, the untreated FPH increased FRAP in a dose-response trend. However, when compared with the FPH from HP pretreated samples at the highest concentration (2.5 mg/mL), none exhibited superior FRAP activity (Figure 2), indicating that HP pretreatment does not ameliorate the FRAP. In addition, as indicated in Figure 2C, the untreated FPH decreased the ABTS radical values in a dose-dependent manner. FPH from samples pretreated at 200 MPa × 4 min, 200 MPa × 8 min, 400 MPa × 4 min, 400 MPa × 8 min, 600 MPa × 4 min, and 600 MPa × 8 min exhibited no significant differences in ABTS radical scavenging compared to the untreated sample (Figure 2D).

Regarding the ORAC assay, the untreated FPH was tested at concentrations of 0.1, 0.5 and 1 mg/mL, while FPH from samples pretreated by HP were compared to the untreated FPH at the second most active concentration (0.5 mg/mL). As depicted in Figure 2E, the untreated FPH scavenged the peroxyl radicals generated by 2,2′-azobis(2-methylpropionamidine) dihydrochloride, achieving the best scavenging capacities at 0.5 and 1 mg/mL. However, no significant difference in ORAC values was observed between untreated FPH and the HP pretreated samples at 0.5 mg/mL (Figure 2F).

In the DPPH assay, the untreated FPH sample inhibited the DPPH radicals in a dose-dependent manner (Figure 2G). In contrast, FPH from samples pretreated at 200 MPa × 4 min, 200 MPa × 8 min, 400 MPa × 4 min, 400 MPa × min, 600 MPa × 4 min, and 600 MPa × 8 min showed no capacity to ameliorate the DPPH radical scavenging activity compared to the untreated FPH sample (Figure 2H). Considering all the in vitro results, FPH is confirmed to be a source of antioxidant peptides. Numerous studies have investigated and analyzed the antioxidant activity of FPH from different species, with bioactivity being directly correlated to the hydrolysis conditions and significantly influenced by the enzyme used for their production. Therefore, the choice of enzyme is crucial for hydrolyzing fish proteins to obtain hydrolysates with enhanced antioxidant potential [29].

However, none of the FPH obtained from samples pretreated by HP demonstrated enhanced antioxidant activity compared to the untreated FPH. While all assays showed that these FPH exhibited antioxidant properties, their activities were comparable to the untreated FPH in the ABTS and ORAC tests, with a slight reduction in the antioxidant activity observed in the FRAP and DPPH assays (Figure 2, see also Table S1 in Supplementary Materials). These findings are in contrast with the results from other studies, where fish side streams from other fish sources were subjected to pressure-assisted enzymatic hydrolysis, resulting in FPH with enhanced antioxidant activity, as evidenced by higher DPPH scavenging activity and stronger reducing power following HP treatments [20,30]. That could be attributed to the simultaneous application of HP with enzymatic hydrolysis on the existing protein isolates, which allowed the enzymes to hydrolyze the proteins during their intermediate state, resulting in peptides with different functional properties [27].

The results of the current study are consistent with existing literature, which highlights the capacity of protein hydrolysates from various fish species to combat oxidative stress by reducing DPPH and ABTS radicals while augmenting FRAP and ORAC [31,32]. The antioxidant assays performed in this study have varying sensitivities and measure distinct mechanisms of in vitro antioxidant activity, evaluating different aspects of the antioxidant potential of FPH samples. The variations observed in the results across the assays may be attributed to the nature of the bioactive peptides, whose antioxidant mechanism is influenced by critical factors such as amino acid composition and peptide length [33,34]. In particular, the FRAP assay, which measures the ability of a sample to reduce ferric ions to ferrous ions, may show elevated antioxidant values due to the presence of bioactive peptides containing a high proportion of amino acids such as histidine, cysteine, and methionine, which facilitate iron reduction. These amino acids are known for their ability to interact with metal ions and exhibit reducing activity [35]. The potential predominance of such amino acids in the peptide mixture could enhance the observed antioxidant activity in the FRAP assay compared to other methods, which focus on different mechanisms such as scavenging free radicals or measuring overall oxygen radical absorbance capacity.

### 2.3. 3-(4,5-Dimethylthiazol-2-yl)-2,5-Diphenyltetrazolium Bromide (MTT) Assay on Human Intestinal Caco-2 Cells

The MTT assay was performed on human intestinal Caco-2 cell to assess their cellular viability after treatment with increasing concentrations of FPH samples (Figure S1 in Supplementary Materials), prior to further cellular experiments. After 48 h of treatment, no impairment in cellular viability was detected for any of the tested samples at concentrations of 0.1, 0.5, 1, and 5 mg/mL, indicating that FPH did not exhibit cytotoxicity within this concentration range.

### 2.4. Western Blot Experiments for the Investigation of Inducible Nitric Oxide Synthase (iNOS) and Nuclear Factor Erythroid 2-Related Factor 2 (NRF-2) Protein Level Modulation by FPH

To investigate the cellular mechanism of antioxidant action, the Western blot analysis was conducted. Given that no differences in direct antioxidant properties were observed between the untreated FPH sample and samples pretreated by HP, the analysis focused on FPH from samples subjected to pressures of 200 MPa, 400 MPa, and 600 MPa for the longest duration (8 min). Their biological activity was then compared with that of the untreated FPH at cellular level in Caco-2 cells.

**Figure 3:**
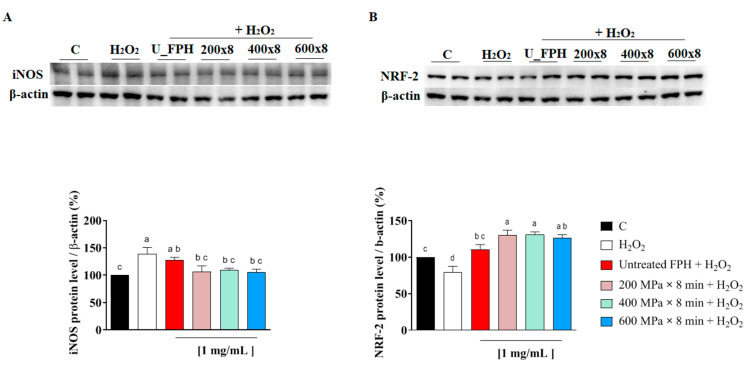
Effect of FPH from the untreated and from samples subjected to HP pretreatment on the H_2_O_2_ (1 mM)-induced iNOS (**A**) and NRF2 (**B**) protein levels in human intestinal Caco-2 cells. Data represent the mean ± SD, *n* = 2 of three independent experiments. Ordinary one-way ANOVA analysis with multiple comparisons was applied. C: control sample (H_2_O). Distinct lowercase letters denote significant differences among the mean values across various treatment groups.

Western blot experiments were exploited to investigate the modulation of two main regulators of oxidative stress; iNOS, involved in the formation of nitric oxide and NRF-2, the key transcription factor that binds to antioxidant response elements situated at the promoters of antioxidant genes, promoting the cellular antioxidant mechanism [36]. As shown in Figure 3 (see also Table S2 in Supplementary Materials), FPH extracted from HP pretreated samples, tested at concentration of 1 mg/mL, significantly decreased iNOS protein levels compared to the prooxidant condition induced by H_2_O_2_, bringing them back to basal conditions (Figure 3A).

Additionally, all tested FPH samples demonstrated a pronounced capacity to enhance NRF-2 production compared to H_2_O_2_-stressed cells. While H_2_O_2_ treatment significantly reduced the NRF-2 production, the untreated FPH restored NRF-2 levels, and FPH from samples pretreated by HP at 200 MPa, 400 MPa, and 600 MPa for 8 min significantly increased NRF-2 protein levels (Figure 3B).

Based on these results, which align with previous research on rainbow trout rest raw material, it can be concluded that peptides derived from the hydrolysis of fish rest raw material are able to exert biological activity, particularly by enhancing the antioxidant profile in the intestinal cellular system [37]. These findings also indicate that, although no significant differences in direct antioxidant activity were observed between the FPH obtained from samples pretreated by HP and the untreated sample, an antioxidant improvement was evident in the Caco-2 system. This could suggest that the application of HP enables the production of hydrolysates with enhanced antioxidant activity.

There are several studies highlighting the antioxidant potential of FPH. The research conducted by Ng, et al. [38] demonstrated the ability of FPH derived from different tilapia tissues to protect against oxidative cellular injury. Similarly, the study carried out by Wang, et al. [39] identified peptides isolated from collagen hydrolysate of redlip croaker (*Pseudosciaena polyactis*) scales, able to reduce the levels of reactive oxygen species (ROS) and malondialdehyde (MDA) while activating intracellular antioxidant enzymes, including superoxide dismutase (SOD), catalase (CAT), and glutathione peroxidase (GSH-Px).

### 2.5. Effect of FPH Peptides on the In Vitro and Cellular DPP-IV Inhibitory Activity

Considering that oxidative stress and hyperglycemia are closely related in the context of metabolic syndrome [40], and that bioactive peptides with antioxidant activity often also act as DPP-IV inhibitors [41], this study investigated the DPP-IV inhibitory activity of untreated FPH and HP pretreated samples, both in vitro and at cellular level.

**Figure 4:**
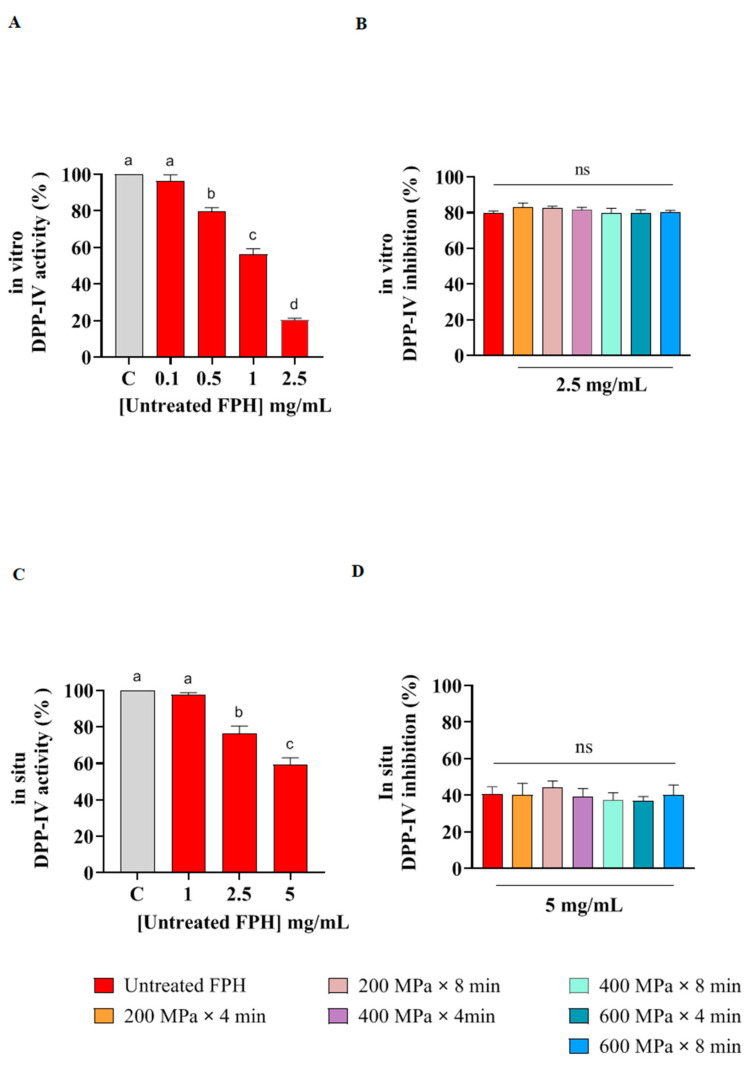
Ability of FPH extracted from the control and from samples subjected to HP pretreatment to inhibit the activity of human recombinant DPP-IV (**A**,**B**) and cellular DPP-IV (**C**,**D**). Data represent the mean ± SD, *n* = 2 of four independent experiments. C: control sample (H2O); Ordinary one-way ANOVA analysis with multiple comparisons was applied. In graphs (**A**,**C**), the untreated sample was compared with vehicle (C) at different concentrations. In graphs (**B**,**D**), FPH from samples pretreated by HP were compared with the untreated at the highest concentration. C: control sample (H_2_O); ns: not significant. Distinct lowercase letters denote significant differences among the mean values across various treatment groups.

For the in vitro evaluations, H-Gly-Pro-AMC was exploited as a substrate to evaluate the inhibitory action of FPH samples against human recombinant DPP-IV. The cleavage of the peptide H-Gly-Pro, catalyzed by DPP-IV, resulted in the release of free AMC group, which allowed for monitoring the enzymatic process at 465 nm. As displayed in Figure 4 (see also Table S3 in Supplementary Materials), untreated FPH reduced the DPP-IV in vitro activity following a dose-response trend, tested at concentrations of 0.1, 0.5, 1, 2.5 mg/mL (Figure 4A). Additionally, the in vitro DPP-IV inhibitory activity of the FPH obtained from HP pretreated samples was compared to that of the untreated sample at the most active concentration (2.5 mg/mL). The results showed that FPH from samples pretreated at 200 MPa × 4 min, 200 MPa × 8 min, 400 MPa × 4 min, 400 MPa × 8 min, 600 MPa × 4 min, and 600 MPa × 8 min inhibited the enzymatic activity of DPP-IV (Figure 4), with no significant difference compared to the untreated FPH (Figure 4B).

Based on these results, FPH from both the untreated and HP pretreated samples were further tested on human intestinal Caco-2 cells, which express the DPP-IV enzyme on their cell membrane surface [42], to verify whether the DPP-IV inhibitory activity of the peptides was preserved at cellular level. As indicated in Figure 4C, the residual in situ DPP-IV activity displayed a dose-dependent trend, with the untreated FPH demonstrating the highest inhibition capacity at 5 mg/mL. Nonetheless, FPH from samples pretreated at 200 MPa × 4 min, 200 MPa × 8 min, 400 MPa × 4 min, 400 MPa × 8 min, 600 MPa × 4 min, and 600 MPa × 8 min inhibited the DPP-IV activity in a comparable manner when tested at 5 mg/mL (Figure 4D). Thus, no statistically significant difference in DPP-IV inhibitory ability was observed between the FPH from the untreated and HP pretreated samples. Considering all the results, the FPH samples were more effective in the assays conducted in vitro than those conducted at cellular level, likely due to physiological metabolism and partial peptide absorption by Caco-2 cells, consistent with our previous work, where the potential antidiabetic activity of peptides derived from rainbow trout rest raw material was explored [37]. Moreover, the application of HPP did not exhibit a modulating impact, as the peptides in the control matrix were already highly active.

Our findings align with studies on other fish matrices such as Atlantic salmon, mackerel, sardine, Pacific hake, and halibut, where the extracted FPH exhibited hypoglycemic activity, particularly through DPP-IV inhibition [15,43,44,45]. Protein hydrolysates extracted from Atlantic salmon was shown to inhibit DPP-IV enzyme with IC50 equal to 1.01 mg/mL [43]. Mackerel protein hydrolysates, obtained using Alcalase and treated with ultrasound at different intensities, reduced the in vitro DPP-IV activity by 73.1% at 2.5 mg/mL when treated at 300 W [44]. This inhibition level is comparable to the FPH from sample pretreated at 200 MPa × 4 min in the current study, which exhibited the highest in vitro DPP-IV inhibitory capacity (82.9%). Furthermore, Rivero-Pino et al. (2020) reported IC50 values ranging from 4 to 8 mg/mL for protein hydrolysates generated from sardine, using a combination of subtilisin, trypsin, and flavourzyme [15]. Another study on fish skin gelatin hydrolysates, produced with flavourzyme, demonstrated DPP-IV inhibitory activities ranging from 45.6% to 48.1% [45].

## 3. Materials and Methods

### 3.1. Preparation of Samples

Fresh Atlantic salmon and rainbow trout rest raw material, including heads, tails, skin, bones, and trimmings, were minced in a ratio of 1:1 w/w. The FPH used in this study was prepared in a previous study where the rest raw materials underwent HPP before enzymatic hydrolysis, as described by Kotsoni, Daukšas, Aas, Rustad, Tiwari and Cropotova [28]. HPP was performed at pressures of 200, 400, and 600 MPa, with hold times of 4 and 8 min, respectively. As detailed in that study [28], hydrolysis was performed in glass vessels, where raw material and warm water (50 °C) were added in a ratio of 1:1 (*w*/*w*), away from direct sunlight, and flushed with nitrogen. Natural antioxidants previously prepared at University of Zagreb [46] were incorporated as a 1:1 (*w*/*w*) mix of oregano and chamomile extracts at a concentration of 1.5 mL/kg of raw material. Subsequently, the enzymes Papain FG and Bromelain 400 GDU/g (both from Enzybel) were added into the vessels in equal proportion, in a total amount of 0.1% (*w*/*w*) (1:1). Hydrolysis was left to proceed for 1 h at 53 °C, followed by enzyme inactivation and centrifugation. The resulting water-soluble FPH from the six high-pressure (HP) pretreated samples and the control (untreated sample) were stored at −80 °C. A portion of the FPH from that study [28] was freeze dried (Freezone 12 L, −84 °C, Labconco Corporation, Kansas City, MI, USA) and held at −80 °C until further analysis.

### 3.2. Molecular Weight Distribution 

100 mg freeze-dried FPH was dissolved in 10 mL Milli-Q (MQ) water. 100 µL of this solution was diluted in 900 µL 10% acetonitrile, to a final concentration of 1 mg/mL hydrolysate. 2 µL of this sample was injected on an AQUITY UPLC H-Class PLUS System (Waters Corporation, Milford, MA, USA) coupled with an AQUITY BEH125 SEC 1.7 μ 4.6 mm × 150 mm column (Waters) and an AQUITY UPLC PDA Detector (Waters Corporation, Milford, MA, USA) set to 220 nm. The isocratic mobile phase consisted of 100 mM sodium phosphate buffer at pH 6.8, with a flow rate of 0.5 mL/min over a duration of 15 min. The column temperature was set to 30 °C for analysis. The peptide profile was determined using a standard curve based on the following eleven peptide standards, purchased from Merck; bovine serum albumin (66,000 Da), cytochrome C (12,327 Da), aprotinin (6500 Da), insulin A (2531 Da), angiotensin II (1046.2 Da), Met-enkephalin (573.7 Da), Leu-enkephalin (555.6 Da), Val-Tyr-val (379.5 Da), Gly-Tyr (238.2 Da), hydroxyproline (131 Da), and L-cystein (121 Da). Chromatograms were manually integrated and separated into intervals of <0.2, 0.2–0.5, 0.5–1, 1–2, 2–5, 5–10, 10–15, and 15–20 kDa, expressed as percentages of the total area. All samples were analysed in triplicate.

### 3.3. FPH Ultrafiltration

FPH were ultrafiltrated using a 3 kDa cut-off Millipore UF System ultrafiltration (UF) membrane (Millipore, Bedford, MA, USA) at 13,300 × g for 20 min, prior to biological activity studies. After being dried in a Speed-Vac (Martin Christ Gefriertrocknungsanlagen GmbH, Osterode am Harz, Germany), the recovered peptide solutions were kept at −80 °C until further analysis.

### 3.4. FRAP Assay

The sample’s capacity to convert ferric ions (Fe^3+^) into ferrous ions (Fe^2+^) was assessed using the FRAP assay [47]. 140 μL of FRAP reagent was mixed with 10 μL of FPH at final concentrations of 0.1, 0.5, 1, 2.5 mg/mL. The FRAP reagent was prepared by combining 13 mL of 0.3 M acetate buffer (pH 3.6), 1.3 mL of 20 mM FeCl_3_ × 6H_2_0, and 1.3 mL of a 10 mM TPTZ (Sigma-Aldrich, Milan, Italy) solution in 40 mM HCl. The microplate was incubated for 30 min at 37 °C, and the absorbance was measured at 593 nm using a Synergy^TM^ HT-multimode microplate reader (Biotek, Bad Friedrichshall, Germany). The Ferric Reducing Antioxidant Power was calculated as:

FRAP (%) = (untreated FPH sample absorbance t30 − untreated FPH sample absorbance t0) × 100)/(C absorbance t30 − C absorbance t0)
or FRAP (%) = (HP pretreated FPH sample absorbance t30 − HP pretreated FPH sample absorbance t0) × 100)/(untreated FPH sample absorbance t30 − untreated FPH sample absorbance t0)(1)

where t0 is the absorbance at at 593 nm at time 0; t30 is the absorbance at 593 nm after 30 min; FPH sample absorbance is the absorbance at 593 nm of FRAP reagent + FPH sample and C absorbance is the absorbance of control (vehicle (H_2_O)) + FRAP reagent at 593 nm.

### 3.5. ABTS Radical Scavenging Activity Assay

The ABTS assay is based on the reduction of the antioxidant-induced 2,2-azino-bis-(3-ethylbenzothiazoline-6-sulfonic acid) radical [47]. 7 mM ABTS solution (Sigma-Aldrich, Milan, Italy) was combined with 2.45 mM potassium persulfate (1:1) to generate the ABTS radical cation (ABTS^+^·), which was then left to stand at room temperature in the dark for 16 h. The ABTS reagent was prepared by diluting the ABTS^+^· with 5 mM phosphate buffer (pH 7.4) to reach a stable absorbance of 0.700 (±0.02) at 730 nm. Then, 140 μL of diluted ABTS^+^· solution was mixed with 10 μL of FPH at final concentrations of 0.01, 0.05, 0.1, and 0.5 mg/mL. The mixture was incubated for 30 min at 30 °C, and the absorbance was measured at 730 nm using a Synergy™ HT-multimode microplate reader (Biotek, Bad Friedrichshall, Germany). The values were calculated using this formula:

ABTS Radical Scavenging activity (%) = (FPH sample absorbance) × 100)/C absorbance(2)

where FPH sample absorbance is the absorbance at 730 nm of ABTS radical + FPH sample and C absorbance is the absorbance of control (vehicle (H_2_O)) + ABTS radical at 730 nm.

### 3.6. ORAC Assay

The ORAC assay utilizes the azo 2,2′-azobis(2-methylpropionamidine) dihydrochloride (AAPH, Sigma-Aldrich, Milan, Italy) to scavenge the produced peroxyl radicals [47]. 50 μL of sodium fluorescein (2.934 mg/L) (Sigma) was mixed with 25 μL of FPH at final concentrations of 0.1, 0.5, 5, and 1 mg/mL, and the mixture was incubated for 15 min at 37 °C. Following incubation, 25 μL of AAPH (60.84 mM) was added, and the decay of fluorescein was monitored using a Synergy™ HT-multimode microplate reader (Biotek, Bad Friedrichshall, Germany) at its maximum emission of 528/20 nm, with readings taken every 5 min for 120 min. The area under the curve (AUC) for each sample was calculated by subtracting the AUC of the blank. The antioxidant capacity was then calculated using a Trolox calibration curve (2–38 μM). The Oxygen Radical Absorbance Capacity was calculated as:

ORAC (%) = (FPH Oxygen Radical Absorbance Capacity) × 100)/(C Oxygen Radical Absorbance Capacity)(3)

where FPH Oxygen Radical Absorbance Capacity is the absorbance of the fluorescein decay of the FPH samples and C Oxygen Radical Absorbance Capacity is the absorbance of the fluorescein decay of control (vehicle (H_2_O) at maximum emission of 528/20.

### 3.7. DPPH Assay

The in vitro antioxidant activity was measured using the DPPH assay, which was carried out using a standard procedure with minor adjustments [47]. 45 μL of 0.0125 mM DPPH solution dissolved in methanol was added at concentrations of 0.1, 1, 5 mg/mL, into 15 μL of FPH. The scavenging reaction of DPPH radicals was performed by incubating the mixture for 30 min at room temperature in the dark. Following the incubation, the absorbance was measured at 520 nm. The ability to scavenge DPPH radical was calculated by the following equation:

DPPH Radical Scavenging activity (%) = (FPH sample absorbance) × 100)/C absorbance(4)

where FPH sample absorbance is the absorbance of DPPH radical + FPH sample at 520 nm and C absorbance is the absorbance of control (vehicle (H_2_O)) + DPPH radical at 520 nm.

### 3.8. Caco-2 Cell Culture

Caco-2 cells, acquired from INSERM (Paris, France), were regularly sub-cultured at 50% density. The cells were kept at 37 °C in an atmosphere of 5% CO_2_ with 25 mM glucose, 3.7 g/L of NaHCO_3_, 4 mM stable L-glutamine, 1% non-essential amino acids, 100 U/L penicillin, and 100 µg/L streptomycin (complete medium), supplemented with 10% heat-inactivated fetal bovine serum (FBS; Hyclone Laboratories, Logan, UT, USA).

### 3.9. MTT Assay

A total of 30,000 Caco-2 cells/well were seeded in 96-well plates. The cells were then treated with 0.1, 0.5, 1, and 5 mg/mL of FPH or H_2_O in complete growth media for 48 h at 37 °C in a 5% CO_2_ atmosphere. Following this, the solvent was aspirated, and 100 μL of filtered 3-(4,5-dimethylthiazol-2-yl)-2,5-diphenyltetrazolium bromide (MTT) solution was added to each well. After a 2-h incubation at 37 °C in a 5% CO_2_ atmosphere, 100 μL of the lysis buffer (8 mM HCl + 0.5% NP-40 in DMSO) was added to each well after aspirating the 0.5 mg/mL solution. The absorbance was measured at 575 nm using a Synergy^TM^ H1 fluorescence plate reader (Biotek, Bad Friedrichshall, Germany) following 5 min of gentle shaking.

### 3.10. iNOS and NRF2 Protein Level Evaluation by Western Blot Analysis

24-well plates were seeded with 150,000 Caco-2 cells/well, and the plates were then incubated at 37 °C in a 5% CO_2_ atmosphere. The day after, cells were exposed to 1 mg/mL of the untreated FPH and FPH from samples pretreated at 200 MPa × 8 min, 400 MPa × 8 min and 600 MPa × 8 min in a full-growth medium for 24 h. Following the treatment, oxidative stress was induced by adding 1.0 mM H_2_O_2_ for 1 h. The cells were then lysed to obtain proteins. Using primary antibodies against iNOS, NRF-2, and β-actin, Western Blot experiments were conducted under previously reported conditions [48].

### 3.11. In Vitro DPP-IV Inhibitory Activity

The experiments were conducted in triplicate using a half-volume 96-well solid plate (white) under previously modified conditions [49]. A total reaction volume of 50 µL was prepared in a microcentrifuge tube, which included 30 µL of 1 × assay buffer (20 mM Tris-HCl, pH 8, containing 100 mM NaCl and 1 mM EDTA), 10 µL of each sample (at the final concentration of 0.1, 0.5, 1, 2.5 mg/mL) and 10 µL of purified human recombinant DPP-IV enzyme. The mixture was then transferred to each well on the plate, followed by the addition of 50 µL of substrate solution (5 mM H-Gly-Pro-AMC) to initiate the reaction. Then, the plate was incubated at 37 °C for 30 min. Fluorescence signals were measured using a Synergy^TM^ H1 fluorescent plate reader (Biotek, Bad Friedrichshall, Germany) at an excitation/emission wavelength of 360/465 nm.

### 3.12. DPP-IV Inhibitory Activity on Human Intestinal Caco-2 Cells

Cells were seeded on black 96-well plates with clear bottoms at a density 50,000 cells/well. The following day, cells were washed once with 100 µL of PBS without Ca^2+^ and Mg^2+^, and treated with FPH at final concentrations of 1, 2.5, and 5 mg/mL for 1 h. Then, 100 µL of Gly-Pro-AMC substrate at a concentration of 25 µM, prepared in PBS without Ca^2+^ and Mg^2+^, were added to each well. The fluorescence signals (excitation/emission wavelengths 350/450 nm) were measured every 1 min for up to 10 min using a Synergy^TM^ H1 fluorescent plate reader (Biotek, Bad Friedrichshall, Germany).

### 3.13. Statistical Analysis

Statistical analysis was conducted using one-way ANOVA, with a significance level set at *p* < 0.05. Mean differences were further identified using Tukey’s Honestly Significant Difference (HSD) test. Statistical analysis for molecular weight distribution was performed using SigmaPlot software, version 14 (Systat Software Inc., San Jose, CA, USA), while the rest of the data analyses were performed using GraphPad Prism 9 (GraphPad Software, La Jolla, CA, USA).

## 4. Conclusions

The applied HPP had a significant effect on the molecular weight distribution of the peptides in the obtained FPH. Low and moderate pressures (200 and 400 MPa) led to a decrease in the proportion of smaller peptides (1–2 kDa) and an increase in larger peptides (2–5 kDa and 5–10 kDa). In contrast, pretreatment at 600 MPa for 4 min produced FPH with a peptide profile similar to the control, characterized by a higher proportion of smaller peptides, likely due to enhanced protein unfolding and increased enzyme accessibility. Regarding antioxidant activity, in vitro assays showed no significant difference between FPH from HP pretreated samples and the untreated. However, the antioxidant activity of FPH from HP pretreated samples was significantly enhanced in human intestinal Caco-2 cells. Additionally, the antidiabetic (DPP-IV inhibition) effect of FPH from HP pretreated samples was not significantly higher than that of the untreated FPH, both in vitro and in Caco-2 cells. These findings provide valuable insights for the potential use of HPP in modifying the bioactivity of FPH for food applications.

## Data Availability

Data are contained within article.

## Supplementary Materials

The following supporting information can be downloaded at: https://www.mdpi.com/article/10.3390/md22120568/s1, Figure S1: Caco-2 cells images taken after 48 h of treatment with different samples. Table S1: In vitro antioxidant assays results. Table S2: Western blot analysis results. Table S3: In vitro and in situ DPP-IV activity assays results.
