# Supplementary material for: Antioxidant Activity and DPP-IV Inhibitory Effect of Fish Protein Hydrolysates Obtained from High-Pressure Pretreated Mixture of Rainbow Trout (Oncorhynchus mykiss) and Atlantic Salmon (Salmo salar) Rest Raw Material
Source: Mar Drugs. 2024 Dec 18;22(12):568. doi: 10.3390/md22120568 (PMC11677631; doi:10.3390/md22120568)
Supplement: Supplementary file 1 [file marinedrugs-22-00568-s001.zip › marinedrugs-3341512-supplementary.pdf]

# Supplementary Material

## Antioxidant activity and DPP-IV inhibitory effect of fish protein hydrolysates obtained from high-pressure pretreated mixture of rainbow trout (*Oncorhynchus mykiss*) and Atlantic salmon (*Salmo salar*) rest raw material

**Elissavet Kotsoni [1,*], Egidijus Daukšas [1], Grete Hansen Aas [1], Turid Rustad [2], Brijesh K. Tiwari [3], Carmen Lammi [4], Carlotta Bollati [4], Melissa Fanzaga [4], Lorenza d'Adduzio [4], Janne Kristin Stangeland [5] and Janna Cropotova [1,*]**

1 Department of Biological Sciences Ålesund, Norwegian University of Science and Technology, 6009 Ålesund, Norway; egidijus.dauksas@ntnu.no (E.D.); graa@ntnu.no (G.H.A.)
2 Department of Biotechnology and Food Science, Norwegian University of Science and Technology, 7034 Trondheim, Norway; turid.rustad@ntnu.no
3 Food Chemistry and Technology Department, Teagasc Food Research Centre, Ashtown, D15 DY05 Dublin, Ireland; brijesh.tiwari@teagasc.ie
4 Department of Pharmaceutical Sciences, Università degli Studi di Milano, Via Luigi Mangiagalli 25, 20133 Milano, Italy; carmen.lammi@unimi.it (C.L.); carlotta.bollati@unimi.it (C.B.); melissa.fanzaga@unimi.it (M.F.); lorenza.dadduzio@unimi.it (L.d.)
5 Møreforsking AS, Borgundvegen 340, 6009 Ålesund, Norway; janne.kristin.stangeland@moreforsking.no
* Correspondence: elissavet.kotsoni@ntnu.no (E.K.); janna.cropotova@ntnu.no (J.C.)

**Table S1.** *In vitro* antioxidant assays results.

| FRAP | | | ABTS | | |
|---|---|---|---|---|---|
| **Sample** | **Concentration** | **Mean ± SD (%)** | **Sample** | **Concentration** | **Mean ± SD (%)** |
| Untreated FPH | 0,1 mg/mL | 189,4±2,078 | Untreated FPH | 0,01 mg/mL | 87,73±1,014 |
| | 0,5 mg/mL | 235,1±5,082 | | 0,05 mg/ml | 62,88±2,746 |
| | 1 mg/mL | 385,3±8,691 | | 0,1 mg/ml | 49,44±1,732 |
| | 5 mg/mL | 702,5±30,13 | | 0,5 mg/ml | 37,91±1,183 |
| 200 MPa x 4 min | 5 mg/mL | 669,5±30,34 | 200 MPa x 4 min | 0,5 mg/ml | 37,65±1,289 |
| 200 MPa x 8 min | 5 mg/mL | 590,7±25,95 | 200 MPa x 8 min | 0,5 mg/ml | 39,96±2,213 |
| 400 MPa x 4 min | 5 mg/mL | 564,9±27,74 | 400 MPa x 4 min | 0,5 mg/ml | 38,93±4,183 |
| 400 MPa x 8 min | 5 mg/mL | 515,4±25,81 | 400 MPa x 8 min | 0,5 mg/ml | 36,12±1,75 |
| 600 MPa x 4 min | 5 mg/mL | 507,5±31,83 | 600 MPa x 4 min | 0,5 mg/ml | 36,37±2,439 |
| 600 MPa x 8 min | 5 mg/mL | 483,9±27,01 | 600 MPa x 8 min | 0,5 mg/ml | 36,37±1,323 |
| **DPPH** | | | **ORAC** | | |
| **Sample** | **Concentration** | **Mean ± SD (%)** | **Sample** | **Concentration** | **Mean ± SD (%)** |
| Untreated FPH | 0,1 mg/mL | 26,65±2,359 | Untreated FPH | 0,1 mg/mL | 67,59±2,073 |
| | 1 mg/mL | 36,98±1,631 | | 0,5 mg/mL | 113,1±0,7929 |
| | 5 mg/mL | 56,86±0,9183 | | 1 mg/mL | 115,8±0,398 |
| 200 MPa x 4 min | 5 mg/mL | 54,58±1,096 | 200 MPa x 4 min | 1 mg/mL | 118,9±3,088 |
| 200 MPa x 8 min | 5 mg/mL | 47,66±0,7846 | 200 MPa x 8 min | 1 mg/mL | 116,6±0,8558 |
| 400 MPa x 4 min | 5 mg/mL | 51,49±0,5595 | 400 MPa x 4 min | 1 mg/mL | 117,2±0,4262 |

| | | | | | |
|---|---|---|---|---|---|
| 400 MPa x 8 min | 5 mg/mL | 47,45±0,9615 | 400 MPa x 8 min | 1 mg/mL | 115,5±3,092 |
| 600 MPa x 4 min | 5 mg/mL | 55,64±0,5073 | 600 MPa x 4 min | 1 mg/mL | 116,9±0,4071 |
| 600 MPa x 8 min | 5 mg/mL | 50,7±0,3504 | 600 MPa x 8 min | 1 mg/mL | 114,9±0,4864 |

**Table S2.** Western blot analysis results.

| Molecular target | Sample | Mean ± SD (%) |
|---|---|---|
| NRF-2 | C | 100±0 |
| | $H_2O_2$ | 79,05±8,367 |
| | Untreated FPH + $H_2O_2$ | 110,6±6,524 |
| | 200 MPa x 4 min + $H_2O_2$ | 130,1±6,858 |
| | 400 MPa x 4 min + $H_2O_2$ | 131,1±3,498 |
| | 600 MPa x 8 min + $H_2O_2$ | 126,2±4,578 |
| iNOS | C | 100±0 |
| | $H_2O_2$ | 138,7±11,97 |
| | Untreated FPH + $H_2O_2$ | 127,9±4,935 |
| | 200 MPa x 4 min + $H_2O_2$ | 106,6±10,33 |
| | 400 MPa x 4 min + $H_2O_2$ | 109,3±3,23 |
| | 600 MPa x 8 min + $H_2O_2$ | 105,2±5,683 |

**Table S3.** *In vitro* and *in situ* DPP-IV activity assays results.

| **DPPIV *in vitro* activity** | | | **DPPIV *in situ* activity** | | |
|---|---|---|---|---|---|
| **Sample** | **Concentration** | **Mean ± SD (%)** | **Sample** | **Concentration** | **Mean ± SD (%)** |
| Untreated FPH | C | 100±0 | Untreated FPH | C | 100±0 |
| | 0.1 mg/mL | 96,39±3,343 | | 0.1 mg/mL | 96,39±3,343 |
| | 0.5 mg/mL | 79,67±2,093 | | 0.5 mg/mL | 79,67±2,093 |
| | 1 mg/mL | 56,29±3,015 | | 1 mg/mL | 56,29±3,015 |
| **DPPIV *in vitro* inhibition** | | | **DPPIV *in situ* inhibition** | | |
| 200 MPa x 4 min | C | 100±0 | 200 MPa x 4 min | C | 100±0 |
| 200 MPa x 4 min | 2.5 mg/mL | 82,92±2,425 | 200 MPa x 4 min | 5 mg/mL | 40,18±6,33 |
| 200 MPa x 8 min | C | 100±0 | 200 MPa x 8 min | C | 100±0 |
| 200 MPa x 8 min | 2.5 mg/mL | 82,84±0,8767 | 200 MPa x 8 min | 5 mg/mL | 44,2±3,66 |
| 400 MPa x 4 min | C | 100±0 | 400 MPa x 4 min | C | 100±0 |
| 400 MPa x 4 min | 2.5 mg/mL | 81,55±1,607 | 400 MPa x 4 min | 5 mg/mL | 39,43±4,257 |
| 400 MPa x 8 min | C | 100±0 | 400 MPa x 8 min | C | 100±0 |
| 400 MPa x 8 min | 2.5 mg/mL | 79,74±2,744 | 400 MPa x 8 min | 5 mg/mL | 37,54±3,815 |
| 600 MPa x 4 min | C | 100±0 | 600 MPa x 4 min | C | 100±0 |
| 600 MPa x 4 min | 2.5 mg/mL | 80,03±1,62 | 600 MPa x 4 min | 5 mg/mL | 37,04±2,223 |
| 600 MPa x 8 min | C | 100±0 | 600 MPa x 8 min | C | 100±0 |
| 600 MPa x 8 min | 2.5 mg/mL | 80,22±1,083 | 600 MPa x 8 min | 5 mg/mL | 40,24±5,486 |

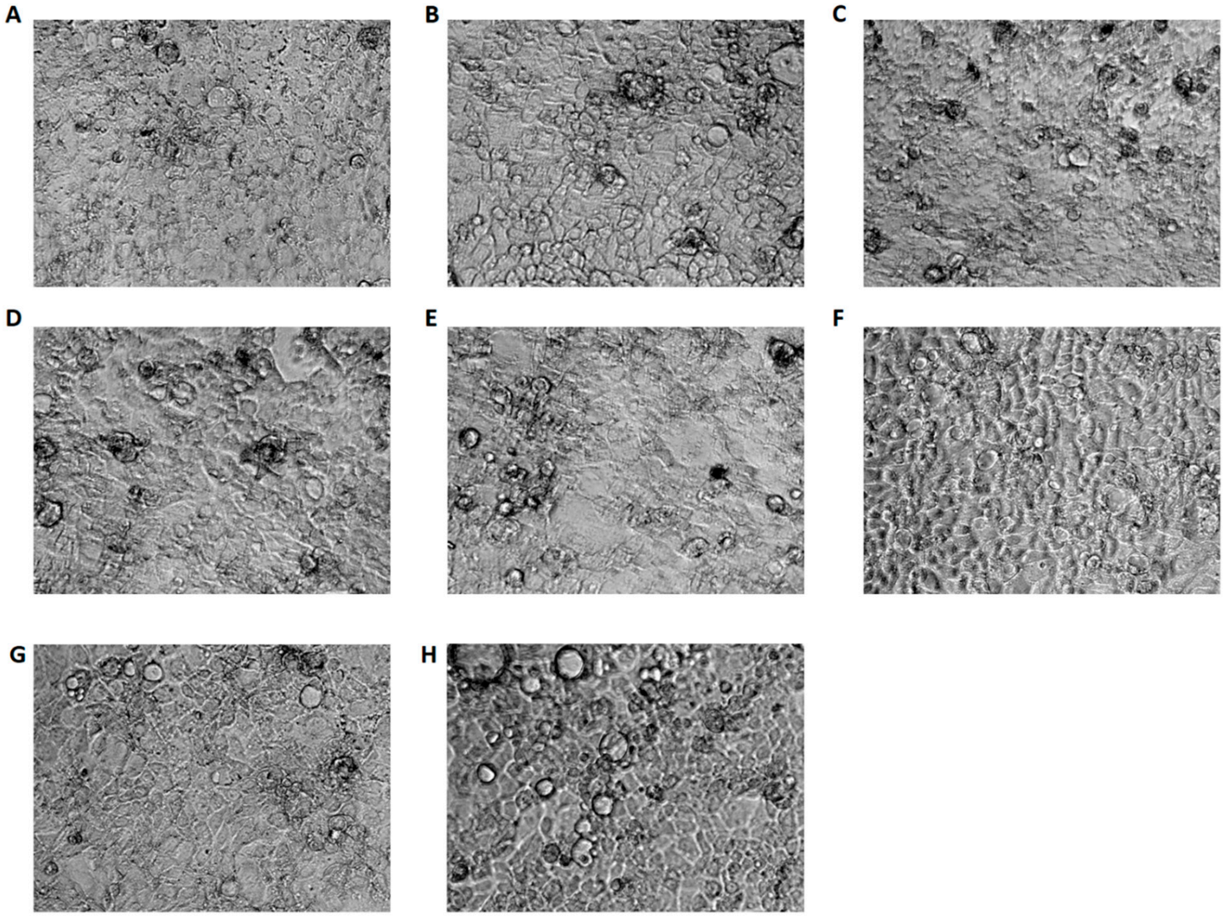


**Figure S1.** Caco-2 cells images taken after 48h of treatment with different samples: (A) $H_2O$, (B) untreated FPH, (C) FPH from sample pretreated at 200MPa x 4 min, (D) FPH from sample pretreated at 200MPa x 8 min, (E) FPH from sample pretreated at 400MPa x 4 min, (F) FPH from sample pretreated at 400MPa x 8 min, (G) FPH from sample pretreated at 600MPa x 4 min, and (H) FPH from sample pretreated at 600MPa x 8 min. Imaging was performed at 20x magnification.
